# Origin of the human malaria parasite *Plasmodium vivax*

**DOI:** 10.1016/j.pt.2024.05.001

**Published:** 2024-05-28

**Authors:** Paul M. Sharp, Lindsey J. Plenderleith, Richard L. Culleton, Beatrice H. Hahn

**Affiliations:** 1 Institute of Ecology and Evolution, University of Edinburgh, Edinburgh EH9 3FL, UK; 2 Centre for Immunity, Infection, and Evolution, University of Edinburgh, Edinburgh EH9 3FL, UK; 3 The Roslin Institute, University of Edinburgh, Midlothian EH25 9RG, UK; 4 Division of Parasitology, Proteo-Science Centre, Ehime University, 454 Shitsukawa, Toon, Ehime 791-0295, Japan; 5 Department of Medicine, Perelman School of Medicine, University of Pennsylvania, Philadelphia, PA 19104, USA; 6 Department of Microbiology, Perelman School of Medicine, University of Pennsylvania, Philadelphia, PA 19104, USA

## Abstract

The geographic origin of *Plasmodium vivax*, a leading cause of human malaria, has been the subject of much speculation. Here we review the evolutionary history of *P. vivax* and *P. vivax*-like parasites in humans and non-human primates on three continents, providing overwhelming evidence for an African origin. This conclusion is consistent with recent reports showing that Duffy-negative humans in Africa are, in fact, susceptible to *P. vivax*, with parasites invading Duffy-antigen-expressing erythroid precursors. Thus, the African origin of *P. vivax* not only explains the distribution of the Duffy-negative genotype but also provides new insight into the history and status of *P. vivax* malaria in Africa and efforts geared toward its eradication.

## Debates about the geographic origin of *P. vivax*

**Figure 1.**
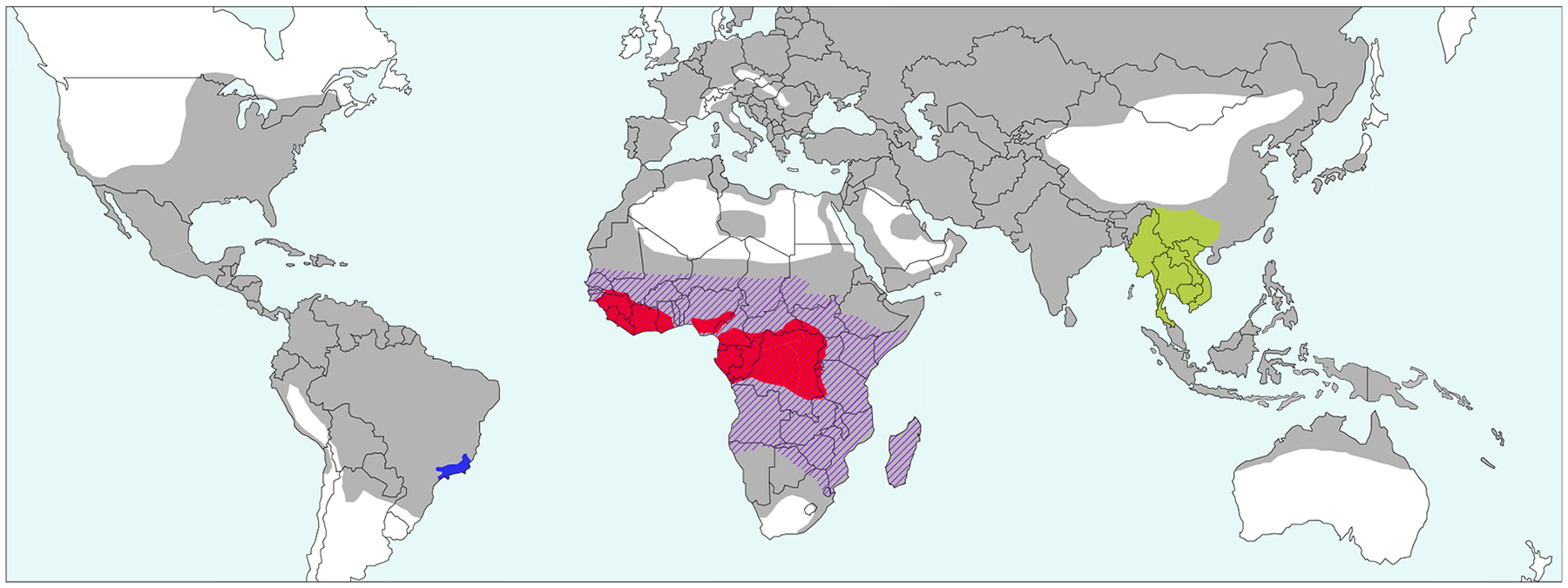
Global distribution of *Plasmodium vivax* and related species found in non-human primates. The approximate areas where human *P. vivax* infections were found over the past few centuries are shown in gray (adapted from [78]). The area where South American monkeys are infected with *Plasmodium simium* is shown in blue [23]. The range of African apes (chimpanzees, bonobos, and gorillas), infected with *P. vivax*-like parasites, is shown in red. The approximate area in southeast Asia previously invoked as the site of origin of human *P. vivax* is shown in green. The area where the Duffy-negative phenotype has a frequency of more than 70% is hatched [62].

*P. vivax* is responsible for an estimated 14 million cases of malaria each year and is the cause of considerable morbidity in humans [1]. It is the most widespread human malaria parasite outside Africa, with a range extending to more temperate regions than the other human malaria parasites, such that about one-third of the human population is at risk of infection (Figure 1). However, across most of sub-Saharan Africa this parasite has appeared to be very rare. This has been interpreted as resulting from the high frequency of a mutation in humans that prevents expression of the **Duffy antigen receptor for chemokines** (**DARC**) (see Glossary) on the surface of red blood cells; DARC is a receptor used by, and apparently essential for, *P. vivax* for invasion of reticulocytes [2,3]. It has generally been assumed that *P. vivax* did not parasitize our ape ancestors but arose from a **zoonotic** source some time during human evolution, perhaps several hundred thousand years ago [4,5]. In this context, the geographical origin of *P. vivax* has been the subject of much speculation, with Asia, Africa, and South America each having been suggested at different times. Consequently, the possible role of *P. vivax* as the agent providing the selective advantage for **Duffy-negativity** in central Africa has been debated; if *P. vivax* did not originate in Africa, it seems necessary to invoke some other, as yet unidentified, cause for the spread of this mutation in humans. Here we show that the geographic origin of *P. vivax* has implications not only for understanding the distribution of the Duffy-negative mutation in humans but also the history and status of *P. vivax* malaria in Africa and the prospects for its eradication.

## Relatives of *P. vivax* infecting non-human primates

Prior to an explosion of work on **African ape** parasites that began about 15 years ago [6], relatives of *P. vivax* had already been found in non-human primates on three continents, sparking a variety of disparate hypotheses about the origin of the human parasite. In southeast Asia, two species, *Plasmodium cynomolgi* and *Plasmodium hylobati*, infecting macaques (*Macaca* species) and gibbons (*Hylobates* species), respectively, had long been recognized as being likely close relatives of *P. vivax* [7]. This was confirmed by DNA sequence data, which also revealed that a large number of other species from Asian macaques, including *Plasmodium coatneyi, Plasmodium* fi*eldi*, *Plasmodium fragile*, *Plasmodium inui*, *Plasmodium knowlesi*, and *Plasmodium simiovale*, also fell within this clade [8–10]. Additional species considered similar to *P. vivax* had been identified in samples from Asian apes, that is, gibbons (*Plasmodium eylesi*, *Plasmodium jeffreyi*, and *Plasmodium youngi*) and orangutans (*Plasmodium pitheci* from *Pongo* species) [7,11], but no sequence data were available to confirm their relationships. In Africa, classic studies starting about 100 years ago had identified one species morphologically indistinguishable from *P. vivax* infecting chimpanzees and gorillas (*Plasmodium schwetzi*), and later a second, much more divergent, species (*Plasmodium gonderi*) was found in several monkey species. In South America, a very close relative of *P. vivax*, termed *Plasmodium simium*, had been found infecting New World monkeys [7].

## Out of South America?

*P. simium* was first identified in 1951 as a parasite infecting howler monkeys (*Alouatta fusca*) in Brazil [7]. Subsequent nationwide surveys [12] found the same parasite in woolly spider monkeys (*Brachyteles* sp.), though much less commonly, and it seems restricted to southeast Brazil (Figure 1). *P. simium* was reported as resembling both *Plasmodium ovale* and *P. vivax* morphologically, but genetic analyses confirmed a very close relationship to *P. vivax* [13]. Recognizing that this close genetic similarity implied a recent host switch between New World monkeys and humans, some authors suggested (‘tentatively’) a direction of transfer from monkeys to humans [14]. Later, when only a small amount of variability at microsatellite loci was found among more than 100 global *P. vivax* isolates, it was suggested that this reflected a recent origin of the human parasite, with the only well-characterized candidate for a source being *P. simium* [15]. As recently as 2011, it was asserted that a New World monkey origin of human *P. vivax* could not be excluded [16]. However, if *P. simium* was the progenitor of *P. vivax*, the emergence from South America must have been post-Columbian, whereas records from Greece in the fifth century BCE (before common era) seem to give a clear description of **tertian malaria**, most likely due to *P. vivax* [17,18].

With further genetic, and especially population genomic, studies it has become clear that *P. simium* exhibits much less genetic diversity than *P. vivax* (Table 1), and that this must reflect transmission from humans to monkeys, probably within the past 500 years [19–21]. While this rules out South American primates as the original source of *P. vivax*, humans in that region have recently experienced zoonotic reintroductions of *P. vivax* from local monkeys. Several outbreaks of vivax malaria in humans in areas of Brazil, where the parasite had previously been eliminated, were shown to reflect back transmissions of *P. simium* from howler monkeys to man [22,23].

## Out of Asia?

**Figure 2.**
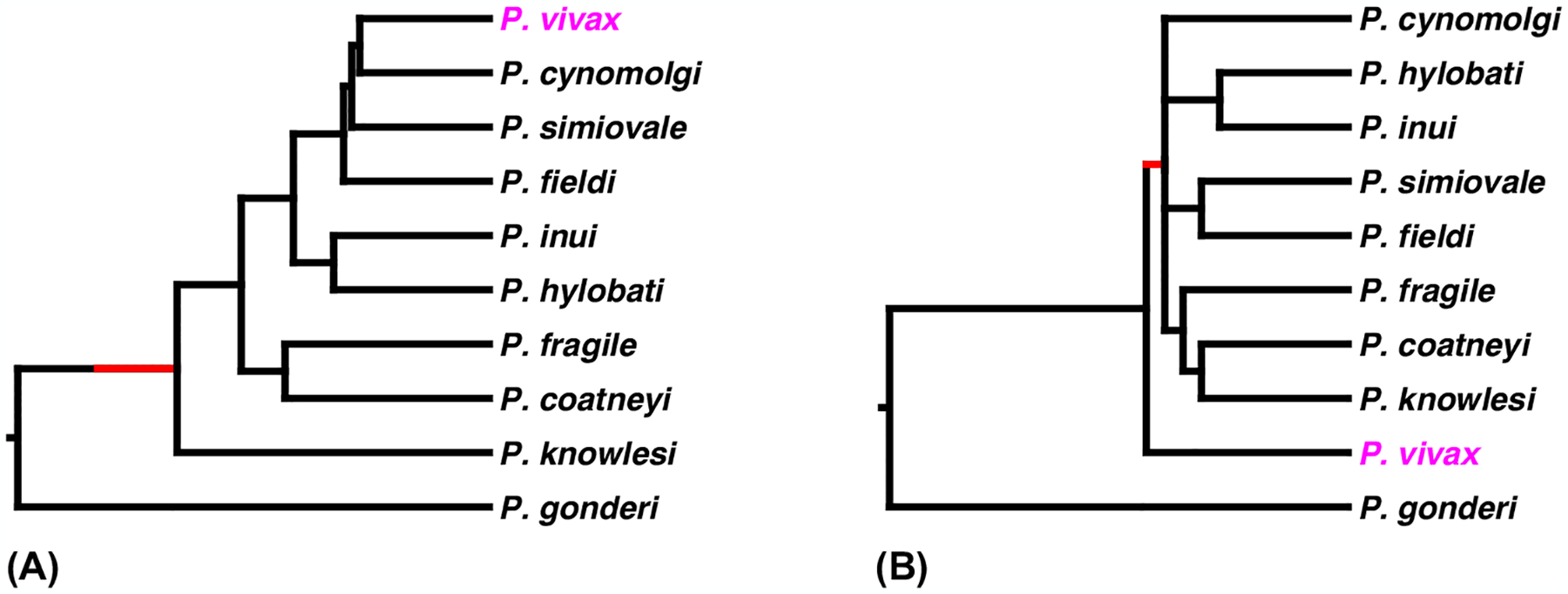
Relationship of *Plasmodium vivax* to southeast Asian parasite species. *P. vivax* from humans is shown in magenta. *Plasmodium gonderi* infects African monkeys; all other *Plasmodium* species shown infect non-human primates in southeast Asia. (A) ‘Traditional’ phylogeny, as based on analysis of a small number of genes from the mitochondrial genome (adapted from [79]); similar trees were shown in [5,9,10,24,25,27]. (B) Revised phylogeny, based on large numbers of proteins encoded by the nuclear genome (adapted from [44]); similar trees were shown in [6,45]; proteins encoded by the apicoplast genome yielded the same topology [43]. In both trees, the red branch indicates the inferred move from hosts in Africa to hosts in southeast Asia.

Citing texts that pre-dated the use of **molecular phylogeny** [7,11], in 1998 it was asserted that ‘it is generally accepted that *P. vivax* originated in Asia’ [9]. As noted earlier, the area of the world with the largest number of different species closely related to *P. vivax* is southeast Asia. In a phylogeny derived from the cytochrome b gene (encoded by the mitochondrial genome) *P. vivax* lay deep within the radiation of six species from Asian primates, pointing to an Asian origin, although bootstrap analyses indicated that the branching order was not statistically reliable [9]. Subsequent analyses, mostly based on full-length mitochondrial DNA (mtDNA) genomes, all confirmed the close relationship between *P. vivax* and the southeast Asian parasites [4,5,10,24,25]. Moreover, most of these studies indicated strong support for the position of *P. vivax* within the radiation of these Asian parasite species (Figure 2A), leading to the conclusion that the human parasite must have originated by zoonotic transmission from a non-human primate in Asia (Figure 1). There was some debate about which of these Asian parasite species was most closely related to, and thus might represent the best model for, or even the source of, *P. vivax*, although the consensus view implicated *P. cynomolgi*, a parasite that infects a number of macaque species across southeast Asia [5,25,26].

To time the emergence of *P. vivax* in humans, molecular clocks have been used to estimate the time of divergence of *P. vivax* from its closest extant relative, and/or the date of the most recent common ancestor (MRCA) of *P. vivax* strains. Depending on the assumptions made to calibrate the clock, the split between *P. vivax* and *P. cynomolgi* has been estimated at 2.1 to 5.4 million years ago [27]. Across various studies, the MRCA of human *P. vivax* was estimated to be between about 50 000 and 300 000 years ago [4,10,24]; at the extreme, the MRCA was estimated at 370 000 years ago (although erroneously reported as 768 000 years ago), by assuming that *P. vivax* has evolved at the same rate as mammals [28]. These studies that pointed to an Asian origin of *P. vivax* have tended to imply that the event was quite ancient, with some authors suggesting a possible zoonotic transfer to *Homo erectus* more than 1 million years ago, with a later genetic bottleneck around the time of the origin of modern *Homo sapiens* [4].

## *P. vivax*-like parasites in African apes

Around 100 years ago, Reichenow (working in Cameroon) and Blacklock and Adler (in Sierra Leone) described a variety of *Plasmodium* parasites in blood samples from chimpanzees and gorillas, with one form being very similar to *P. vivax* and *P. ovale* (summarized in Chapter 12 in [7]). Subsequent work, involving failed attempts at cross-infection, suggested that the ape parasite was a distinct species, and it was named *P. schwetzi* [29], but it remained unclear which of the two human parasites this species was more similar to. No material remained from this early work for genetic characterization, perhaps explaining why the finding of this ape parasite in Africa had little impact on the consensus view that the *P. vivax* lineage originated in Asia.

The perspective on *P. schwetzi*, and more generally on *P. vivax* in Africa, started to shift in 2010 when several groups reported the detection of *P. vivax*-like sequences in samples from African apes. Krief *et al*. initially reported the presence of such sequences in the blood of two apes, one a captive eastern chimpanzee (*Pan troglodytes schweinfurthii*) in a sanctuary near Kampala in Uganda, and the other an orphan central chimpanzee (*Pan troglodytes troglodytes*) rescued in the Democratic Republic of Congo (DRC) [30]. However, since these authors also found drug-resistant strains of *Plasmodium falciparum* of human origin in some of their study apes, it was unclear whether these *P. vivax*-like sequences came from parasites that infected apes in the wild. Soon after, similar sequences were found in fecal samples from one wild eastern chimpanzee (in the DRC) and three wild western lowland gorillas (*Gorilla gorilla*) from Cameroon and the Central African Republic [31], as well as in tissues from a dead wild western chimpanzee (*Pan troglodytes verus*) in Cote d’Ivoire [32]. These findings hinted that *P. vivax* was widespread among African apes, and prompted more directed investigations. Prugnolle *et al*. subsequently amplified *P. vivax*-like sequences from the blood of two chimpanzees and two gorillas, and also from a mosquito (*Anopheles moucheti*), all sampled in Gabon [33], while our own noninvasive studies identified similar sequences in more than 70 fecal samples from chimpanzees, western (*G. gorilla*) and eastern lowland gorillas (*Gorilla beringei*) collected at more than 30 field sites across central Africa [34]. More recently, we found *P. vivax*-like sequences in four samples from bonobos (*Pan paniscus*) at one site in the east of their range [35]. Detection of parasites in fecal samples is substantially less sensitive than in blood [34], and so these findings indicate that the four species of African apes represent a substantial reservoir of *P. vivax*-like parasites across sub-Saharan Africa (Figure 1).

**Figure 3.**
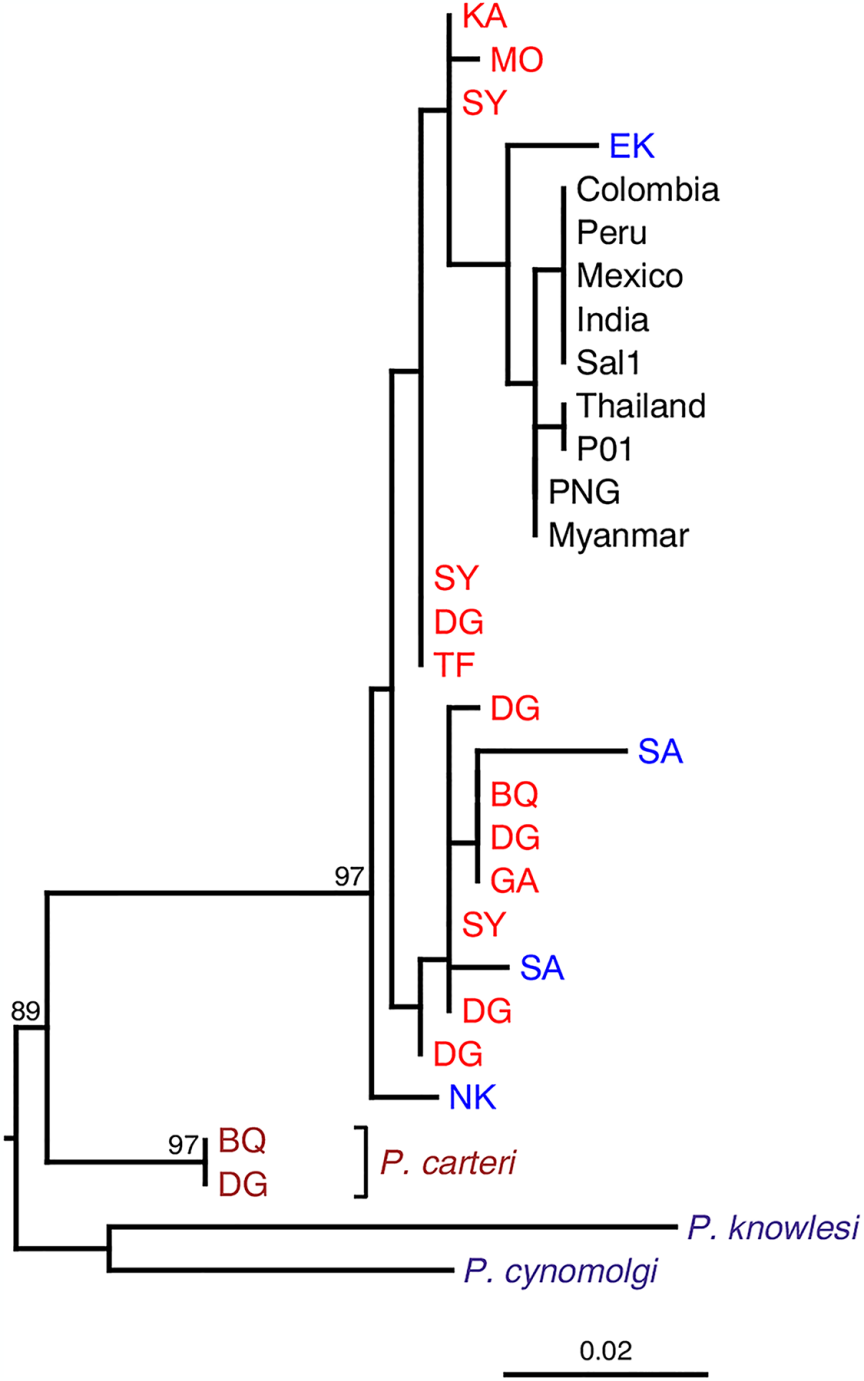
Relationship of human *Plasmodium vivax* to *P. vivax*-like and *Plasmodium carteri* parasites from African ape samples. The evolutionary tree contains *P. vivax* sequences from humans (black), *P. vivax*-like sequences from chimpanzees (red) and gorillas (blue), as well as two sequences (purple) from a divergent species (termed *P. carteri*) also found in chimpanzee samples. Two species from macaques in southeast Asia (*Plasmodium cynomolgi* and *Plasmodium knowlesi*) were used to root the tree according to Figure 2B in the main text. Two-letter codes for ape sequences denote different sample site locations. This tree (adapted from [36]) was derived from gene PVP01_1418600; in trees derived from other genes, human *P. vivax* strains always cluster, but the relationship between *P. vivax* and *P. vivax*-like strains varies due to recombination.

There were additional important observations. First, phylogenetic analyses showed that the human parasites formed a **monophyletic clade** either (dependent on the gene) within the radiation of, or as a sister clade to, the ape parasites; by contrast, there was no clear separation between parasite sequences derived from different ape species (Figure 3) [33,34,36,37]. Second, it became apparent that the ape parasites were genetically much more diverse than human *P. vivax* strains from around the globe. Analyzing genome sequences, we found nucleotide sequence diversity at selectively neutral sites to be more than ten times higher in the ape parasites (Table 1) [36].

This finding of *P. vivax*-like parasites infecting apes in Africa seemed to present a substantial new problem: if it is still accepted that *P. vivax* must have originated in southeast Asia, because of the phylogenetic argument (Figure 2A), how did infection reach apes in central Africa, and why is the genetic diversity among the ape parasites so much greater than that among the human parasites? Prugnolle *et al*. explored four hypothetical scenarios in an attempt to explain this, but none seemed plausible or sufficient to fully explain the observations [33].

## Out of Africa!

Further insight into this debate about the origin of *P. vivax*, and its ultimate resolution, has come from two additional observations. First, two chimpanzee samples from different field sites in Cameroon contained DNA sequences that were closely related to, but nevertheless clearly distinct from, *P. vivax* [34]. Sequences of mitochondrial, **apicoplast**, and nuclear DNA from these two samples consistently clustered as an outgroup to *P. vivax*, indicating a new species more closely related to *P. vivax* than are any of the southeast Asian primate parasites (Figure 3) [38]. Since these sequences have so far been detected in only two samples, we cannot exclude the possibility that this species does not infect apes. We recently found DNA sequences from a porcupine parasite, *Plasmodium atheruri*, as well as porcupine mtDNA, in a fecal sample from a human hunter–gatherer, indicating consumption of *Plasmodium*-infected bushmeat [39]. Thus, it is possible that this new species found in two chimpanzee samples normally infects another host that is hunted by chimpanzees. Alternatively, this new species may infect chimpanzees but yield low blood parasitemia levels, making its detection difficult. Whatever the true host of this new species, it has so far been found only in central Africa. The most parsimonious interpretation of this is clearly that the common ancestor of *P. vivax*-like parasites from apes and this newly identified species existed in Africa, indicating that the lineage that gave rise to *P. vivax* has been in Africa for a long time. In recognition of Richard Carter’s sustained advocacy of the idea that *P. vivax* had an African origin [40,41], we have previously suggested the name *Plasmodium carteri* for this new species [38].

Second, it has become apparent that the original phylogenetic arguments pointing to an Asian origin of *P. vivax* were ill-founded [42]. An analysis of 30 proteins encoded by the apicoplast genome showed *P. vivax* as the basal, or first diverging lineage, outside the clade of southeast Asian parasite species [43]. The same result (Figure 2B) was obtained analyzing proteins encoded by 627 genes from nuclear chromosomes 1–4 [43], and in our own analysis of 1693 proteins encoded by orthologous genes from across all 14 chromosomes [6]. More recent analyses of 2773 proteins compared across the entire mammalian *Plasmodium* clade [44], or 4436 proteins from the clade of *P. gonderi*, *P. vivax*, and the southeast Asia parasites [45], have also confirmed that *P. vivax* lies outside the radiation of the southeast Asian species. These studies have shown that the phylogenies derived from previous analyses of mtDNA sequences, or a limited number of other sequences, were misleading.

The earliest diverging branch among the relatives of *P. vivax* is that of *P. gonderi* (Figure 2), a parasite that infects monkeys in Africa. Thus, it is now clear that the ancestral lineage leading to *P. vivax* need never have left Africa, and that the ancestor of the southeast Asian clade most likely migrated to Asia after its divergence from the common ancestor of *P. vivax*, *P. vivax*-like and *P. carteri* (Figures 2B and 3); this move to Asia may have been with the ancestor of the current Asian macaque species, around 4–5 million years ago [46].

## Genetic diversity in *P. vivax* from humans

Despite overwhelming evidence to the contrary, some authors have continued to argue that an Asian origin of *P. vivax* cannot be excluded [47–51]. One argument points to the extent of genetic diversity observed among *P. vivax* strains from different countries. If a species has recently emerged from a point of origin and spread through a series of founder events that successively reduced levels of genetic variation we can expect, at least initially, a negative correlation between genetic diversity and distance from that origin. Modern humans first appeared in East Africa and spread from there to Asia at least 50 000 years ago [52]. Consistent with this, our species exhibits reduced genetic diversity with increasing migration distance from East Africa [53]. Similarly, the human malaria parasite *P. falciparum* was found to show a negative correlation between diversity and distance from central Africa, when strains from sites in Africa, southeast Asia, and Oceania were compared [54]. Again, this is consistent with the known African origin of *P. falciparum*, because it emerged from the zoonotic transmission of a gorilla parasite [31]. By contrast, *P. vivax* appears to exhibit reduced genetic diversity with increasing distance from a putative origin in southeast Asia. In a comparison of samples from 14 regions, Daron and colleagues found nucleotide diversity to be highest in the Greater Mekong region (Thailand, Cambodia, and Myanmar), lower in China, India, Borneo, and New Guinea, and especially low in Papua New Guinea, and central and South America [47]. Two African sites showed moderate (Ethiopia) or very low (Mauritania) diversity. There was a strong negative correlation between genetic diversity and great circle distance from a location in the Greater Mekong region, which was interpreted as reflecting the same process previously invoked to explain the geographic structure of diversity in *P. falciparum* [47].

However, over time, any initial pattern of reduced genetic diversity with increasing distance from an origin will be disrupted by subsequent demographic effects, and eventually diversity will reflect the intensity of transmission and the recent effective population size of the local parasite population. Two lines of evidence indicate that *P. vivax* has infected humans over a much longer time-scale than *P. falciparum*. First, genetic diversity is higher in *P. vivax* than in *P. falciparum* [6,28], despite a potentially slower rate of molecular evolution due to periods of time spent dormant, as **hypnozoites** in the liver [55]. Second, the Duffy-negative mutation appears to have spread in humans much earlier than mutations conferring resistance to *P. falciparum* malaria [56]. Thus, while diversity in *P. falciparum* may still reflect its recent out-of-Africa origin, diversity in *P. vivax* may now be dominated by more recent events. For example, human antimalaria interventions have had enormous, but region-specific, impacts on parasite populations. The WHO’s Global Malaria Eradication Programme (GMEP), which ran from 1955 to 1969, resulted in huge reductions in parasite prevalence in some regions [57]. The GMEP was particularly successful in India, where cases of malaria (all species) were reduced from an estimated 75 million in 1947 to just under 50 000 in 1961 [58]. This reduction of the parasite population can explain why genetic diversity of *P. vivax* in India is reduced compared with that in countries of the Greater Mekong region. Generally low genetic diversity of *P. vivax* in Africa could be explained by the spread of the Duffy-negative mutation eliminating most strains in the past, so that today’s populations in Africa largely result from reintroduction from Asia [59]; it is also likely that genetic diversity in Africa, especially Mauritania, was underestimated due to limited sampling [60]. Low diversity among central and South American strains reflects an origin within the past 500 years, albeit sourced from perhaps four different ancestral populations [61].

## *P. vivax* infection of Duffy-negative individuals

Over the past 15–20 years there have been sporadic, but increasing numbers of, reports of *P. vivax* across the regions of west and central Africa where the Duffy-negative allele is near fixation [62] (Figure 1). In most instances these infections have been submicroscopic, or characterized by very low levels of parasitemia, requiring PCR amplification of parasite sequences from the blood as evidence of infection [63–65]. By 2022 the accumulation of such reports led Baird to conclude that, contrary to existing dogma, there is ‘widespread transmission of *P. vivax* in Duffy-negative Africa’ [3]. Noting that there were likely many *P. vivax*-infected individuals with undetectable parasitemia in the peripheral blood, Baird suggested various scenarios by which transmission could nevertheless be efficient enough to maintain endemicity in Duffy-negative populations [3].

Initially it was unclear whether only particular strains of *P. vivax*, harboring specific mutation(s), and perhaps utilizing an alternative receptor, were able to infect Duffy-negative individuals. However, in Sudan and Ethiopia, where both Duffy-negative and Duffy-positive individuals are common, characterization of eight microsatellite loci revealed no obvious sign of genetic differentiation between *P. vivax* strains infecting the two types of host [66]. Moreover, two groups have recently independently demonstrated that the Duffy-negative mutation does not completely abrogate expression of the DARC protein, but limits it to tissue-resident erythroid progenitor cells [67,68]. This transiently expressed DARC receptor was found to be fully functional and able to mediate *P. vivax* invasion, thus negating the need to invoke an alternative invasion pathway in Duffy-negative individuals [67,68].

A shift of the parasite biomass away from the peripheral blood to sites of active erythropoiesis, such as bone marrow, in Duffy-negative people suggests that many of their *P. vivax* infections have previously been missed. Thus, this parasite may have persisted across west and central Africa, even after the spread of the Duffy-negative mutation. All currently available *P. vivax* genome sequences from this region appear to be derived from populations that were reintroduced from Asia, although only very few strains from west and central Africa have been characterized [69,70]. Thus, the possibility that remnants of ancient African human *P. vivax* lineages still infect some Duffy-negative individuals cannot be excluded.

## Concluding remarks

The evidence supporting an African origin for *P. vivax* is now overwhelming. Furthermore, given that (i) all species within the *Laverania* subgenus (comprised of *P. falciparum* and related parasites from apes [31,35]) are found in Africa, (ii) the closest known relatives of *Plasmoidum malariae* and of both species in the *P. ovale* complex are also in African apes [6,71], and (iii) members of the *Vinckeia* subgenus of *Plasmodium*, found in rodents, bats and ungulates, also originate from Africa, it now seems very likely that the common ancestor of all these mammalian *Plasmodium* lineages was in Africa. This runs counter to the traditional view, prior to the advent of molecular data, that most primate malarias originated in Asia (Chapter 1 in [7]). Other than parasites carried by humans migrating in recent times, the only mammalian *Plasmodium* lineage that likely evolved in Asia is that comprised of *P. knowlesi*, *P. cynomolgi*, etc., to which *P. vivax* is closely related; however, it is now apparent that the migration to Asia almost certainly occurred after the divergence of this lineage from the ancestor of *P. vivax* (Figure 2B).

Despite this resolution of the issue of origin, at least three interesting, and interrelated, questions about the evolution of *P. vivax* remain. First, what is the past, and ongoing, relationship between *P. vivax* and the Duffy-negative mutation? A commonly voiced question is whether selection due to *P. vivax* infection would have been sufficient to raise the frequency of the Duffy-negative mutation to near fixation, given that *P. vivax* infections are rarely lethal. While non-fatal, *P. vivax* infections nevertheless can cause severe illness, and it has recently been estimated that the Duffy-negative mutation has a selection coefficient of about 8% [72]. A mutation conferring a fitness advantage of as little as 8% to its carriers could increase from a frequency of 1% to 99% in fewer than 250 generations (Chapter 3 in [73]). Thus, *P. vivax* could easily have selected for the spread of the Duffy negative allele in less than 10 000 years. However, given that until very recently *P. vivax* seemed extremely rare in humans across central Africa, and Duffy-negativity may result in **neutropenia** [74], the question has been what maintains the high frequency of the mutant allele? Since *P. vivax*-like parasites from apes can infect Duffy-positive humans [33], it has been tempting to speculate that exposure to such strains via mosquitoes that have bitten apes could have provided the continued selection pressure [6] (see Outstanding questions). However, the increasing evidence that *P. vivax* is endemic among Duffy-negative individuals provides a more direct explanation. Nevertheless, it will be interesting to gauge the extent of exposure of humans to the ape parasites, to judge how much of a zoonotic threat they could pose.

Second, what exactly is the relationship between *P. vivax* in humans and *P. vivax*-like parasites in African apes? The observation that there is no consistent phylogenetic separation between strains infecting chimpanzees and gorillas indicates that, in contrast to species in the *Laverania* clade [31], these parasites are less host-specific. Moreover, it is apparent that ape parasites can infect Duffy-positive humans [33]. Thus, it is possible that, prior to the spread of the Duffy-negative mutation, *P. vivax* may have infected all African ape species, including humans. Later, the initial spread of the Duffy-negative mutation could have nearly eliminated *P. vivax* from humans in sub-Saharan Africa. Then the continued existence of human *P. vivax* resulted from a parasite lineage that escaped Africa, either when modern humans first did so (at least 50 000 years ago [52]), or at some later date. The greatly reduced diversity of human, compared with ape, *P. vivax* can be explained if this escape from Africa involved a severe genetic bottleneck [6,36]. Alternatively, if there was in the past some barrier, such that ape parasites were not normally transmitted to humans, the bottleneck may have occurred at the point of origin of human *P. vivax*, via zoonotic transmission. Either scenario is consistent with phylogenetic analyses of individual genes which often show the human *P. vivax* clade as lying within the radiation of ape strains, especially when sequences representing the full diversity of ape strains, and sequences from a close outgroup (such as *P. carteri*) are available (Figure 3) [36]. Sequences of some genes show the ape and human strains as sister clades, indicating that some lineage sorting has occurred within the ape strains, subsequent to the divergence of the human parasite lineage. As yet, there has been no report of more recent introgression from ape *P. vivax*-like into human *P. vivax*.

Third, now that we know that *P. vivax* is endemic across the Duffy-negative areas of Africa, the question arises whether any of the strains now found in Africa represent remnants of the original *P. vivax* that circulated among humans in Africa prior to the spread of the mutation, or whether all represent subsequent reintroductions from the stock that had escaped out-of-Africa (see Outstanding questions). In some regions, such as Mauritania in West Africa, where the Duffy-negative allele has not reached a high frequency, and there appears to have been long-established *P. vivax* transmission [60], it may seem unlikely that this parasite species was ever eliminated. By contrast, *P. vivax* in Madagascar is likely to have been reintroduced from Asia, because the indigenous people of Madagascar arrived from southeast Asia and east Africa within the past few thousand years [75]. For strains from central Africa, it will be necessary to characterize a sufficient number of parasite genomes to ascertain whether they all cluster within the global human (out-of-Africa) *P. vivax* clade. It will be of particular interest to examine postmortem tissue samples such as bone marrow, spleen, and liver, to determine the true prevalence and nature of *P. vivax* strains among Duffy-negative individuals (see Outstanding questions).

The eradication of malaria continues to be an aspiration [76]. In this context, it is important to understand the circumstances surrounding the origins of each of the human malaria parasites, in order to gauge the extent of future zoonotic risk. Much less attention has been paid to *P. vivax* compared with *P. falciparum* in Africa, and the resultant paucity of information has potentially undermined attempts at malaria elimination [77]. It is also unclear to what extent *P. vivax*-like parasites in apes might thwart any program for eradication: would ape parasites need to adapt to become highly transmissible in Duffy-positive humans, and are the ape strains capable of infecting Duffy-negatives? The conclusion that *P. vivax* originated in Africa, from a pool of parasites that still exists in African apes, is highly pertinent to these questions.

## Figures and Tables

**Table 1.** Levels of genetic diversity among strains of human *Plasmodium vivax*, ape *P. vivax*-like parasites, and *Plasmodium simium* from New World (NW) monkeys

Parasite	Hosts	Π^a^	Π^b^	Π^c^	Π-4^d^
*P. simium* ^e^	NW monkeys	0.00013	0.00016	Not available (NA)	NA
*P. vivax*	Humans	0.00079	0.00056	0.00085	0.00121
*P. vivax*-like	African apes	NA	NA	0.00698	0.01604

a Genetic diversity is estimated as Π, the average pairwise difference per nucleotide site. Diversity at all aligned genomic sites [20]. *P. vivax* value is from a global sample of parasites.

b Diversity at all aligned genomic sites [19]. The *P. vivax* value given is for parasites from Brazil; values for samples from Peru, Columbia, and Mexico ranged from 0.00052 to 0.00062.

c Diversity at all sites within genes [36]. *P. vivax* value is from a global sample of parasites.

d Diversity at fourfold degenerate (presumably selectively neutral) sites in genes [36]. *P. vivax* value is from a global sample of parasites.

e The *P. simium* values includes strains isolated from humans after zoonotic transmission from monkeys.

## Highlights

The evolutionary relationships among *Plasmodium vivax* in humans, *P. vivax*-like parasites in African apes, and other related parasites infecting non-human primates indicate that *P. vivax* originated in Africa, and can explain why the human parasites exhibit much less genetic diversity.

The geographic origin of *P. vivax* provides an explanation for the emergence of the Duffy-negative mutation among humans in Africa.

The recent discovery that *P. vivax* can invade erythroid precursor cells of Duffy-negative humans suggests that remnants of an ancient *P. vivax* lineage that infected both humans and apes before the spread of the Duffy-negative mutation may still circulate in Africa.

A more extensive characterization of *P. vivax* in Duffy-negative individuals, focusing in particular on parasites sequestered in hematopoietic tissues, is urgently needed.

## Outstanding questions

Exactly how common is *P. vivax* infection among Duffy-negative individuals across west and central Africa, and how often is *P. vivax* transmitted from Duffy-negative hosts?

Did *P. vivax* initially need to evolve to infect Duffy-negative hosts?

Is there any ancestral *P. vivax*, from prior to the spread of the Duffy-negative mutation, remaining in humans in central Africa?

Does ape *P. vivax*-like contribute selection pressure to maintain the Duffy-negative genotype?

Why has a Duffy-negative mutation not spread among African apes?

Does *P. carteri* appear to be rare in apes because of low level parasitemia and fecal shedding?

## Glossary

African ape: chimpanzees, bonobos, and (two species of) gorillas are the African apes, and are the closest extant evolutionary relatives of humans
Apicoplast: an organelle in *Plasmodium* species (and in many other members of the phylum Apicomplexa) ancestrally derived from an algal chloroplast that contains a small genome (of about 35 kb)
Duffy antigen receptor for chemokines (DARC): a molecule expressed on the surface of circulating red blood cells, used by *P. vivax* to invade those cells
Duffy-negativity: a condition of humans in which DARC is absent from the surface of red blood cells due to a mutation in the promoter of the gene encoding DARC
Hypnozoites: dormant, that is, non-replicating, stages in the life cycle of *P. vivax* associated with latency and relapse following reactivation
Molecular phylogeny: comparison of DNA or protein sequences to estimate the evolutionary relationships among species; the evolutionary tree produced by this approach
Monophyletic clade: a group comprised of all descendants of a particular common ancestor
Neutropenia: abnormally low levels of neutrophils, a form of white blood cell involved in fighting infections
Tertian malaria: malaria occurring with a periodicity of 48 h
Zoonotic: transmitted to humans from animals
